# *In silico* Analysis of Acyl-CoA-Binding Protein Expression in Soybean

**DOI:** 10.3389/fpls.2021.646938

**Published:** 2021-04-15

**Authors:** Nur Syifaq Azlan, Ze-Hua Guo, Wai-Shing Yung, Zhili Wang, Hon-Ming Lam, Shiu-Cheung Lung, Mee-Len Chye

**Affiliations:** ^1^School of Biological Sciences, The University of Hong Kong, Pokfulam, Hong Kong; ^2^School of Life Sciences and Center for Soybean Research of the State Key Laboratory of Agrobiotechnology, The Chinese University of Hong Kong, Shatin, Hong Kong

**Keywords:** abiotic stress, acyl-CoA-binding protein, biotic stress, *Glycine max*, lipid trafficking, microarray, transcriptomics, protein structure

## Abstract

Plant acyl-CoA-binding proteins (ACBPs) form a highly conserved protein family that binds to acyl-CoA esters as well as other lipid and protein interactors to function in developmental and stress responses. This protein family had been extensively studied in non-leguminous species such as *Arabidopsis thaliana* (thale cress), *Oryza sativa* (rice), and *Brassica napus* (oilseed rape). However, the characterization of soybean (*Glycine max*) ACBPs, designated GmACBPs, has remained unreported although this legume is a globally important crop cultivated for its high oil and protein content, and plays a significant role in the food and chemical industries. In this study, 11 members of the GmACBP family from four classes, comprising Class I (small), Class II (ankyrin repeats), Class III (large), and Class IV (kelch motif), were identified. For each class, more than one copy occurred and their domain architecture including the acyl-CoA-binding domain was compared with Arabidopsis and rice. The expression profile, tertiary structure and subcellular localization of each GmACBP were predicted, and the similarities and differences between GmACBPs and other plant ACBPs were deduced. A potential role for some Class III GmACBPs in nodulation, not previously encountered in non-leguminous ACBPs, has emerged. Interestingly, the sole member of Class III ACBP in each of non-leguminous Arabidopsis and rice had been previously identified in plant-pathogen interactions. As plant ACBPs are known to play important roles in development and responses to abiotic and biotic stresses, the *in silico* expression profiles on GmACBPs, gathered from data mining of RNA-sequencing and microarray analyses, will lay the foundation for future studies in their applications in biotechnology.

## Introduction

Soybean (*Glycine max*) is one of the most important global grain crops and plays a very prominent role in the food industry, because of its high protein (∼40%) and oil content in its seeds (∼20%) (Assefa et al., 2019). Soybean oil accounted for 362 of 596 million metric tons of total global oilseed production (George, 2021). In some parts of the world, especially in Asia, many types of dried and fermented food are derived from soybean including soy sauce, soy milk, and tofu (Barrett, 2006). Furthermore, the production of soybean meal processed from soybean as a nutritious protein-rich food for livestock and poultry (Pantalone, 2012) totals 246.05 million metric tons globally (Soybean meal: world supply and demand, 2020). Soybean also supports several chemical industries related to the production of biodiesels, bioplastics and cosmetics (Gaonkar and Rosentrater, 2019).

Similar to many other important crops, soybean is subject to environmental challenges that disrupt growth and development which lead to reductions in yield and quality. A major problem faced in agriculture is salinity because excessive amounts of salt (exceeding 40 mM NaCl) in soils adversely affect the physiological and biochemical processes in plants (Miransari, 2016). Most legumes including soybean are sensitive to salinity (Hasanuzzaman et al., 2016) posing a severe threat to the soybean industry as land contaminated by high salt expands following saline intrusion by rising sea-water levels (FAO, 2018). Also, soybean is sensitive to cold environments of below 10°C that retard vegetative growth (Hasanuzzaman et al., 2016) as its optimal growth temperature ranges from 22.0 to 24.0°C (Choi et al., 2016). In soybean, water availability is especially crucial during the reproductive stage, particularly at seed filling (Desclaux et al., 2000), when seeds accumulate reserves of carbohydrate, protein and lipids (Goldberg et al., 1989). Drought and heat stress at the seed-filling stage are known to alter seed content, including lipid composition (Sehgal et al., 2018). During severe drought, reduction in soybean seed oil along with decrease in the percentages of linoleic and linolenic acids was reported, compromising on the quality of the seed oil (Dornbos and Mullen, 1991).

Besides abiotic stress factors, soybean is vulnerable to biotic stress. Soybean rust is one of the most threatening soybean diseases that affects production in the two biggest producers, United States and Brazil (Yorinori et al., 2005; Langenbach et al., 2016). In southern China, the loss suffered from soybean rust in the 1990s was estimated to be around 20 to 30 million US$/year (Li et al., 2010). Caused by a pathogenic fungus, *Phakopsora pachyrhizi*, this disease became more imminent as soybean *R* gene resistance responses were overcome and fungicide insensitivity escalated (Langenbach et al., 2016). Root and stem rot produced by an oomycete pathogen known as *Phytophthora sojae* is another soybean disease that causes million US$ losses in the United States (Tyler, 2007). An understanding of the molecular aspects underlying each of the abiotic and biotic stresses encountered will provide better strategies to enhance the crop value of soybean.

Lipids are organic molecules that can accomplish functions related to stress signaling and protection in many cellular processes. Besides their roles in response to stress and development (Colin and Jaillais, 2020; Huby et al., 2020; Rogowska and Szakiel, 2020; Wan et al., 2020), they are important in the formation of membranes for compartmentalization of cells and organelles (Goñi, 2014). Waxes and suberin which can be found on the epidermis and endodermis, respectively, participate in defense to protect against pathogenic attack (Seigler, 1998). The *de novo* synthesis of fatty acid (FA) in plants occurs in the plastids after which lipid biosynthesis takes place *via* the ‘prokaryotic’ or ‘eukaryotic’ pathways (Ohlrogge and Jaworski, 1997). In the ‘prokaryotic’ pathway, FA are made and utilized within the plastids while for the ‘eukaryotic’ pathway they must first be exported out of the plastids to the endoplasmic reticulum (ER) for further modifications and integration in lipid assembly (Ohlrogge and Jaworski, 1997). Hence, proteins which transfer lipids play an important role in the mobilization of lipids/FA within the cell (Li et al., 2016). While FATTY ACID EXPORT1 (FAX1) is a membrane protein responsible for the delivery of FA across the chloroplast inner membranes (Li et al., 2015), acyl carrier proteins (ACPs) bind to fatty acyl intermediates during fatty acid synthesis (Mofid et al., 2002). The utilization of FA outside plastids in many cellular pathways will require them to be esterified to acyl-CoA esters by long-chain acyl-CoA synthetase (LACS) (Shockey et al., 2002). It has been reported that candidates for transfer of acyl-CoA esters from the plastids to the ER include the acyl-CoA-binding proteins (ACBPs) (Du et al., 2016).

Acyl-CoA-binding proteins comprise a protein family that share a highly conserved acyl-CoA-binding (ACB) domain of about 80–90 residues (Burton et al., 2005). ACBPs are found in animals, plants, fungi, some eubacteria and archaebacteria (Burton et al., 2005; Islinger et al., 2020). Besides having a canonical role in channeling acyl-CoA esters within subcellular components, their roles can be very diverse in both plants and animals, given the presence of adjoining domains or motifs (Lung and Chye, 2016; Islinger et al., 2020). In plants, ACBPs can be grouped into four classes as dictated by molecular mass and the presence of other functional domain, ankyrin repeats or kelch motif (Du et al., 2016). Besides acyl-CoA esters, to which ACBP classes show different binding affinities, ACBPs also bind to phospholipids (Lung and Chye, 2016). Studies conducted in identifying their subcellular localization as well as their protein interactors have revealed that the roles of ACBPs include mediating stress responses and plant development (Du et al., 2016). This *in silico* study summarizes tissue-specificity and stress-responsiveness of soybean ACBPs, as extracted from data available in SoyBase^1^ and the Soybean eFP Browser^2^. Together with the predicted tertiary structure and subcellular localization, this work provides a foundation in understanding GmACBPs.

## Materials and Methods

### Classification and Homology Modeling of ACBP Homologs in Soybean

Soybean ACBPs (GmACBPs) were identified by BLASTp search using query protein sequences from each class of *Arabidopsis thaliana* (thale cress). Accession numbers of GmACBPs were retrieved from the Phytozome v12.1 database^3^. The GmACBPs were then classified by characterizing their domain architecture. Conserved domains used in the classification of plant ACBPs, namely the acyl-CoA-binding domain (cd00435), ankyrin-repeat domain (cd00204) and kelch motif (pfam01344, pfam07646, pfam13415, pfam13418, and pfam13854) in GmACBP protein sequences were identified by performing an NCBI protein BLAST search^4^ together with the ACBP protein sequences of Arabidopsis and *Oryza sativa* (rice). The domain arrangement and their boundaries were annotated by aligning them with the sequences from the Conserved Domain Database (CDD)^5^. Homology modeling in tertiary structure of GmACBPs was predicted using online tools, Phyre2^6^ (Kelley et al., 2015) and SWISS-MODEL^7^ (Waterhouse et al., 2018). The predicted 3D structures were viewed using NCBI iCn3D^8^ (Wang J. et al., 2020).

### Multiple Protein Sequence Alignment and Sequence Identity of Each Class ACB Domain Between Arabidopsis, Rice and Soybean

Protein sequences of the ACB domain from each class of Arabidopsis ACBPs, rice ACBPs, and soybean ACBPs were aligned using *ClustalW* in MEGA X (Kumar et al., 2018). Geneious Prime version 2021.0.1 software^9^ was used for viewing the alignment and generating the sequence identity between Arabidopsis, rice and soybean.

### Subcellular Localization Prediction of GmACBPs

Subcellular localization of GmACBPs was predicted using TargetP 1.1^10^ (Emanuelsson et al., 2000) and PSORT for plant sequences^11^ (Nakai and Horton, 1999). For both online tools, the prediction was performed using the protein sequences from each GmACBP retrieved from Phytozome v12.1 database (see text footnote 3).

### Data Mining of *GmACBP* Expression in Different Organs and in Response to Stress

*GmACBP* expression data in young leaves, flowers, nodules, roots, developing seeds, and pod shells at different ages were retrieved from the RNA-seq transcriptomic data (Severin et al., 2010) available online at the Soybean eFP Browser (see text footnote 2). Data on the GmACBP expression was derived by using the soybean gene model ID from the Wm82.a1 assembly (Schmutz et al., 2010).

Information on *GmACBP* expression in response to abiotic and biotic stresses was retrieved from SoyBase (see text footnote 1) under SoyBase Expression Explorer^12^. The website hosts expression profiles of soybean gene models that include numerous tissues and organs at different developmental stages from various experiments and publications (Grant et al., 2010). Expression data on each *GmACBP* was explored by inputting the respective gene ID in the provided search bar and clicking on ‘Display Expression.’ RNA-seq and microarray experiments related to the gene of interest would then be displayed. Subsequently, specific *GmACBP* expression profiles in response to abiotic and biotic stresses were collected and tabulated.

For abiotic stress, *GmACBP* expression was analyzed in response to salinity, dehydration and cold stress. The *GmACBP* expression profile in response to salinity and dehydration stress was retrieved from a study using soybean cultivar Williams 82 (Belamkar et al., 2014). A high saline environment was created by placing young soybeans in 100 mM NaCl solution (Belamkar et al., 2014). For dehydration stress, the plants were exposed to air with reduced water availability (Belamkar et al., 2014). Root tissues harvested at 0, 1, 6, and 12 h were used for generating the expression profiles in both stress situations (Belamkar et al., 2014).

For cold stress, the *GmACBP* expression profile was adapted from the RNA-seq data generated from Yamasaki and Randall (2016) and Robison et al. (2019). The seedlings were left at 4°C until harvest at 0, 1 and 24 h (Robison et al., 2019).

The *GmACBP* expression profile in response to *P. pachyrhizi* was plotted based on the microarray data generated by Gregory Alvord et al. (2007) in which two different strains of *P. pachyrhizi* were used, the avirulent Hawaii 94-1 and virulent Taiwan 80-2 (Gregory Alvord et al., 2007). The *GmACBP* expression profile in response to *P. sojae* was retrieved from microarray analysis performed using isolate PT2004C2.S1 (Wang et al., 2010). Inoculation was conducted by Wang et al. (2010) at 24, 48, 72, and 120 h, each with an independent mock-treatment. For sample collection at 24 and 48 hpi (hours post inoculation), the sample was harvested directly at the inoculation site. At 72 and 120 hpi, the samples were taken 7.5 mm below and above the lesion margin of the seedlings (Wang et al., 2010).

### Quantitative Real-Time PCR Analysis of *GmACBPs* in Soybean Root Nodules

Nodule samples and nodular RNA were prepared as previously described (Wang Q. et al., 2020) in which surface-sterilized *Glycine max* cv. C08 seeds were germinated in autoclaved vermiculite. *Sinorhizobium fredii* CCBAU45436 was used for inoculation at 4 days after sowing. Seedlings were watered with Milli-Q water at 3-day interval and grown at 28°C under a 16 h/8 h light-dark cycle. Nodules were harvested at 28 days post-inoculation. Samples were snap-frozen with liquid nitrogen and stored at −80°C before use. Total RNA was extracted with TRIzol reagent (Thermo Fisher, United States) and treated with DNase I, Amplification Grade (Thermo Fisher, United States) following manufacturer’s instructions and cDNA was generated using High-Capacity cDNA Reverse Transcription Kit (Thermo Fisher, United States) with 18-mer oligo dT. The cDNA was diluted 10-fold with Milli-Q water before use in qPCR.

Quantitative real-time PCR (qRT-PCR) was performed using TB Green *Premix Ex Taq* II (Tli RNaseH Plus) (TaKaRa Bio, United States) with Real-Time PCR Detection System, Bio-Rad CFX96 Touch. The program was carried out as follows: 95°C at 30 s, then 95°C at 5 s and 60°C for 30 s with 40 cycles. The experiment was conducted in two biological replicates and the calculation of relative expression level performed using the 2^–ΔΔCt^ method (Livak and Schmittgen, 2001) with normalization to the housekeeping gene *F-BOX PROTEIN2* (Libault et al., 2008). The average of 2^–ΔΔCt^ and standard error of mean, *n* = 3 from two biological replicates were used to plot the graph by using stripped roots (the remaining roots after nodule excision) as the control following (Wang Q. et al., 2020). Gene-specific primers used are listed in Supplementary Table 1.

### Statistical Analysis

Analyses of *GmACBP* expression were carried out using the Student’s *t*-test to determine any significant differences between means.

### Accession Numbers

All the sequence information used in this work was retrieved using the following identifiers: Arabidopsis *AtACBPs* (At5g53470, At4g27780, At4g24230, At3g05420, At5g27630 and At1g31812), rice *OsACBPs* (LOC_Os08g06550.1, LOC_Os06g02490.1, LOC_Os03g37960.1, LOC_Os04g58550.1, LOC_Os03g14000.1 and LOC_Os03g61930.1), soybean *GmACBPs* (Glyma.09g214500, Glyma.04g122900, Glyma.04g233600, Glyma.06g131200, Glyma.01g191900, Glyma.11g050200, Glyma.14g087600, Glyma.17g236700, Glyma.03g236500, Glyma.19g234400, and Glyma.20g235500), and *F-BOX PROTEIN2* (Glyma.02G273700).

## Results

### Classification and Homology Modeling of GmACBPs

**FIGURE 1:**
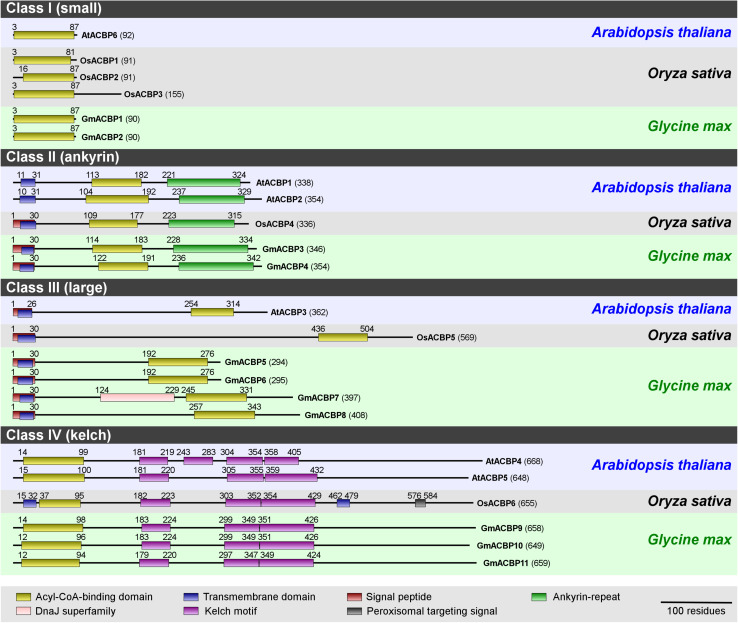
Domain architecture of ACBPs from Arabidopsis, rice, and soybean. ACBP protein sequences from Arabidopsis (*Arabidopsis thaliana*), rice (*Oryza sativa*), and soybean (*Glycine max*) were submitted to the NCBI protein BLAST search (http://blast.ncbi.nlm.nih.gov/Blast.cgi) to identify conserved domains, and their boundaries are annotated by alignment with the Conserved Domain Database (CDD) collection including acyl-CoA-binding domain (cd00435), ankyrin-repeat domain (cd00204) and kelch motif (pfam01344, pfam07646, pfam13415, pfam13418, and pfam13854). Residue numbers are indicated in parentheses. This schematic diagram was modified from Lung and Chye (2016).

Eleven identified GmACBPs were grouped by protein sequence alignment into four classes, based on domain architecture and molecular mass following the classification of the Arabidopsis and rice homologs (Figure 1). The 11 putative GmACBP homologs included two Class I small ACBPs (i.e., GmACBP1 and GmACBP2), two Class II ankyrin-repeat ACBPs (i.e., GmACBP3 and GmACBP4), four Class III large ACBPs (i.e., GmACBP5, GmACBP6, GmACBP7 and GmACBP8) and three Class IV kelch-ACBPs (i.e., GmACBP9, GmACBP10, and GmACBP11).

Each soybean Class I ACBP (GmACBP1 and GmACBP2) consists of 90 amino acids with a single ACB domain similar to Arabidopsis AtACBP6 (Figure 1). In each of Class II GmACBP3 and GmACBP4, a signal peptide absent in Arabidopsis Class II ACBPs, was detected within the transmembrane domain (Figure 1) similar to rice Class II OsACBP4. Class III GmACBP5 and GmACBP6 were more related in protein size and domain arrangement to each other than to GmACBP7 and GmACBP8 which are the largest amongst these four. Each of them contains a signal peptide and a transmembrane domain at the *N*-terminus (Figure 1). Interestingly, GmACBP7 possesses an additional DnaJ superfamily domain, which is absent in rice and Arabidopsis (Figure 1). Class IV GmACBP9, GmACBP10 and GmACBP11 showed conservation to Arabidopsis and rice Class IV ACBPs with the ACB domain at *N-*terminus and kelch motifs in the middle of the protein sequence.

**FIGURE 2:**
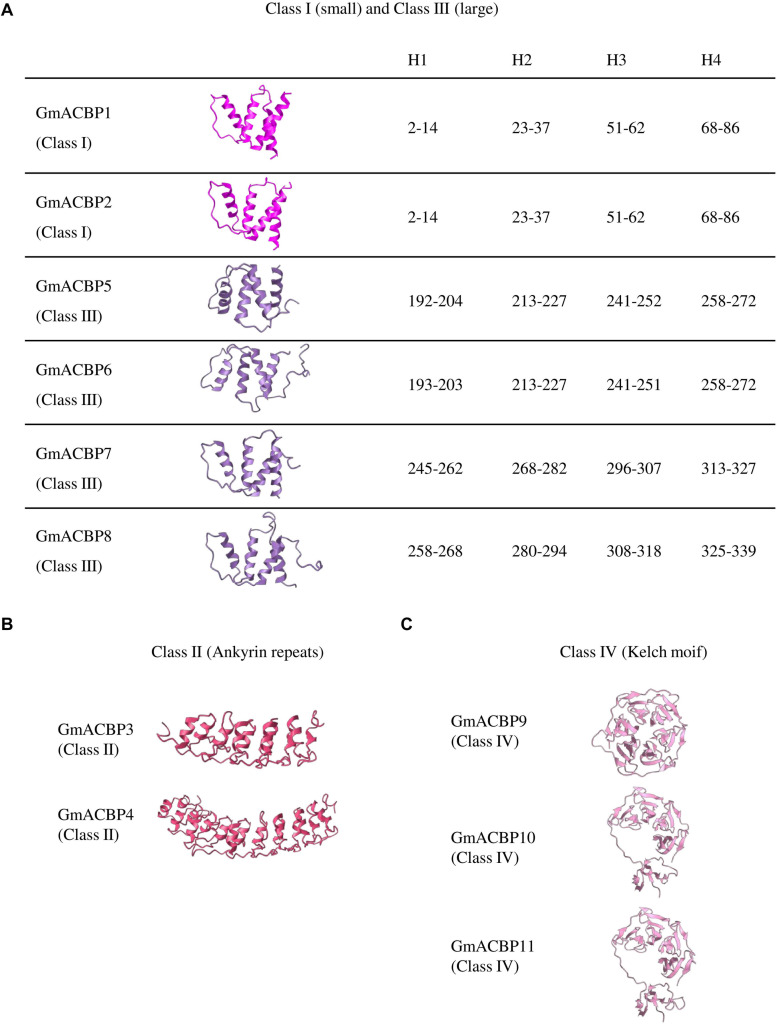
Predicted tertiary structure of GmACBPs. The model was generated by using Phyre2 and SWISS-MODEL. **(A)** Class I and III GmACBPs with their corresponding position of the amino acid residues in the formation of the four helical structures, H1, H2, H3, and H4. **(B)** Predicted 3D model for Class II GmACBPs and their structure included the ankyrin-repeat regions. **(C)** 3D structure of Class IV GmACBPs predicted from SWISS-MODEL, including the region of kelch motif.

Homology modeling in tertiary structure prediction of GmACBPs revealed that each of Class I and Class III GmACBPs consists of four α-helices at the ACB domain (Figure 2A). For Class I GmACBP1 and GmACBP2 the ACB domain was located within amino acid residues 3 to 87 while for Class III GmACBP5 and GmACBP6, the ACB domain spanned from residues 192 to 276 (Figures 1, 2A). The helical structure in Class III GmACBP7 was predicted to be within residues 245 to 327, and the ACB helices in Class III GmACBP8 occurred at residues 258 to 339 (Figure 2A). For Class II GmACBPs, the predicted 3D model (Figure 2B) corresponded to the region of ankyrin repeats at residues 228 to 334 in GmACBP3 and residues 238 to 342 in GmACBP4 (Figure 1). The tertiary structures of Class IV GmACBP9, GmACBP10 and GmACBP11 (Figure 2C) were modeled against the kelch-motif-containing Arabidopsis nitrile-specifier protein (Zhang et al., 2017).

### Conservation at the ACB Domain Within Arabidopsis, Rice, and Soybean Classes

**FIGURE 3:**
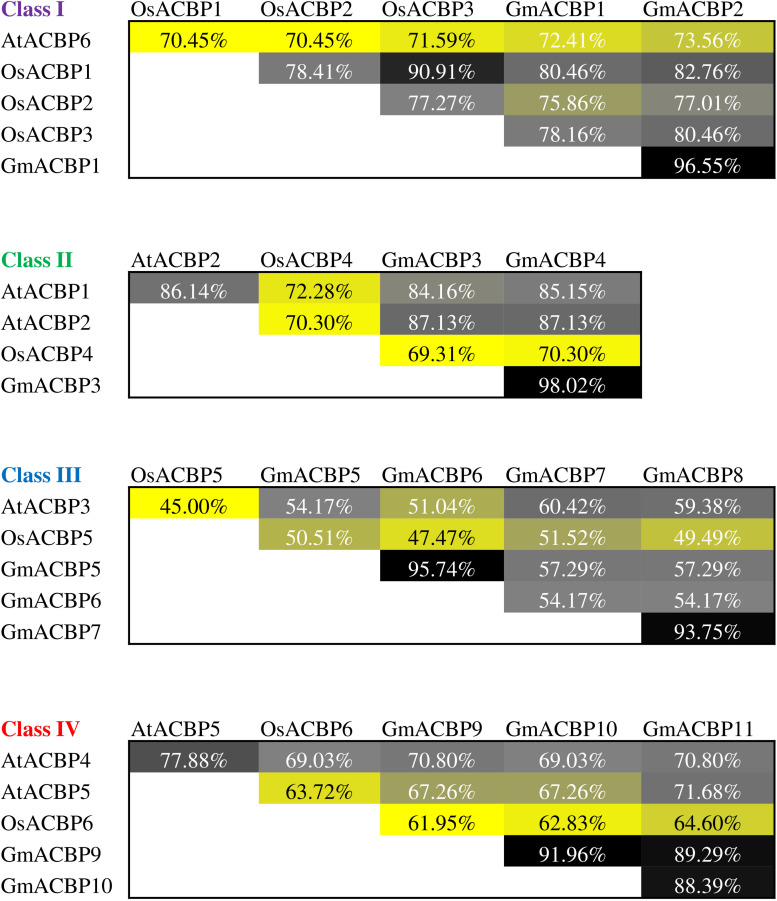
Protein sequence identity of ACB domain in Arabidopsis, rice, and soybean ACBPs within the same classes. Percentage of identical residues was obtained by sequence alignment of the ACB domain in ACBPs from different species using *ClustalW* in MEGA X. The identity values are presented in heatmap in a descending order from black, gray, grayish yellow to yellow.

Multiple sequence alignment of ACB domain of Class I ACBPs from Arabidopsis, rice and soybean revealed that GmACBP1 and GmACBP2 shared higher sequence identity with rice than Arabidopsis (Figure 3). GmACBP1 and GmACBP2 displayed higher identity to OsACBP1, at 80.46% and 82.76% respectively, than the other two Class I OsACBPs (Figure 3). GmACBP1 shared 72.41% identity while GmACBP2 shared 73.56% identity to Arabidopsis AtACBP6 (Figure 3). Class II GmACBP3 and GmACBP4 showed higher identity at the ACB domain with Arabidopsis, especially AtACBP2 with 87.13% identity in comparison to rice (69.31% and 70.30%, respectively) (Figure 3). For Class III, alignment at the ACB domain showed that the sequence identity amongst Arabidopsis, rice, and soybean was below 70% (Figure 3). Among the four members of soybean Class III, GmACBP7 shared highest identity with AtACBP3 and OsACBP5 at 60.42% and 51.52%, respectively (Figure 3). In the alignment of the ACB domain of Class IV, GmACBPs showed higher identity to Arabidopsis than rice, with GmACBP9 and GmACBP10 more similar to AtACBP4, and GmACBP11 to AtACBP5 (Figure 3).

### Subcellular Localization Prediction of GmACBPs

Subcellular localization of GmACBPs was predicted by TargetP 1.1 (Table 1) and PSORT (Table 2). For TargetP 1.1, all proteins were predicted to be localized either at the chloroplast, mitochondria, secretory pathway or any other location. Based on the highest score in TargetP 1.1, Classes I and IV GmACBP were predicted not to be sorted to the chloroplast, mitochondria or secretory pathway while Classes II and III GmACBPs were predicted to be targeted to the secretory pathway (Table 1). Using PSORT, Classes I and IV GmACBPs were deemed cytosolic while Class II GmACBPs scored highest for the plasma membrane (Table 2). For Class III, PSORT predicted that GmACBP5 would most certainly localize outside the cell, while GmACBP6 was predicted to be destined to the vacuole (Table 2). Class III GmACBP7 and GmACBP8 were both predicted to be targeted to the ER membrane (Table 2).

**TABLE 1:** Subcellular localization prediction of GmACBPs by TargetP 1.1.

GmACBP	Chloroplast	Mitochondrion	Secretory pathway	Other
GmACBP1	0.136	0.109	0.102	**0.872**
GmACBP2	0.150	0.103	0.115	**0.838**
GmACBP3	0.009	0.036	**0.981**	0.036
GmACBP4	0.008	0.046	**0.974**	0.036
GmACBP5	0.005	0.046	**0.986**	0.030
GmACBP6	0.007	0.049	**0.987**	0.025
GmACBP7	0.010	0.013	**0.961**	0.096
GmACBP8	0.004	0.013	**0.954**	0.181
GmACBP9	0.025	0.326	0.044	**0.668**
GmACBP10	0.077	0.343	0.041	**0.488**
GmACBP11	0.084	0.275	0.045	**0.539**

*Value is certainty score. Highest score marked in bold indicates that the prediction is most certain to be in that location.*

**TABLE 2:** Subcellular localization prediction of GmACBPs by PSORT.

	Cy	Mit		PM	ER		GB	Out	Vac	Chl (TM)	Mic (Px)
		MtS	IM		M	L					
GmACBP1	**0.650**	0.100									
GmACBP2	**0.650**	0.100									
GmACBP3				**0.811**	0.640	0.100	0.370				
GmACBP4				**0.460**	0.100	0.100		0.100			
GmACBP5					0.100	0.100		**0.820**	0.445		
GmACBP6					0.100	0.100		0.820	**0.868**		
GmACBP7			0.100	0.100	**0.600**					0.302	
GmACBP8			0.100	0.100	**0.600**					0.302	
GmACBP9	**0.650**	0.100									
GmACBP10	**0.450**	0.100								0.100	0.321
GmACBP11	**0.450**	0.100								0.100	0.321

*Value is certainty score. Highest score marked in bold indicated the prediction is most certain to be in that location. Cy, cytoplasm; Mit, mitochondria; MtS, matrix space; IM, inner membrane; PM, plasma membrane; ER, endoplasmic reticulum; M, membrane; L, lumen; GB, Golgi body; Out, outside; Vac, vacuole; Chl, chloroplast; TM, thylakoid membrane; Mic, microbody; Px, peroxisome.*

### *GmACBP* Expression Profiles Across Various Organs of Soybean

**FIGURE 4:**
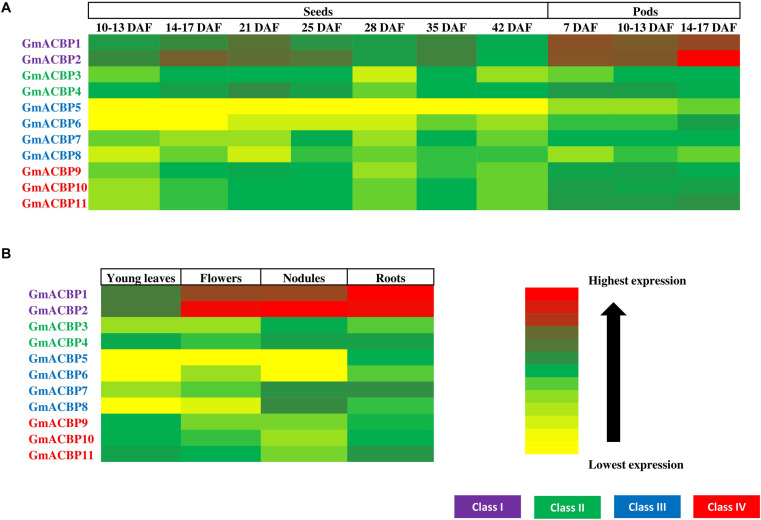
*GmACBP* expression in different organs in the form of a heatmap generated using Microsoft Excel 2010. **(A)** Developing seeds and pods. **(B)** *GmACBP* expression in young leaves, flowers, nodules, and roots. The data were retrieved from RNA-seq analysis performed by Severin et al. (2010) from the Soybean eFP Browser (http://bar.utoronto.ca/efpsoybean/cgi-bin/efpWeb.cgi). The expression level was calculated using RPKM (Severin et al., 2010). The cultivar used was an introgression of *Glycine soja* (PI468916) and *Glycine max* (A81-356022) (Severin et al., 2010). The data were generated by pooled samples of three plants by Severin et al. (2010). Harvested samples by Severin et al. (2010) were based on the ontology terms described in SoyBase Soybean Ontologies (https://www.soybase.org/ontology.php) as follow: young leaves (SOY:0000252), flower (SOY:0001277), nodule (SOY:0001301), roots (SOY:0001183), 10–13 DAF seeds (SOY:0001290), 14–17 DAF seeds (SOY:0001290), 21 DAF seeds (SOY:0001291), 25 DAF seeds (SOY:0001291), 28 DAF seeds (SOY:0001291), 35 DAF seeds (SOY:0001292), 42 DAF seeds (SOY:0001293), 7 DAF pods (SOY:0001280), 10–13 DAF pods (SOY:0001281), and 14–17 DAF pods (SOY:0001282). *GmACBP* classes are shown in different colors.

Figure 4A shows *GmACBP* expression in developing seeds and Class I *GmACBP1* and *GmACBP2* exhibited elevated expression in all stages of seed development while Class III *GmACBP5* and *GmACBP6* have the lowest expression throughout. Class I *GmACBP1* and *GmACBP2* showed similar pattern in expression starting from 28 days after fertilization (DAF) (Figure 4A). Class II *GmACBP3* and *GmACBP4* displayed distinctive patterns of expression throughout seed development. For *GmACBP3*, its expression was maintained at the same level from 14 to 17 DAF until 25 DAF (Figure 4A). For *GmACBP4*, its expression peaked at 21 DAF and was constant from 35 to 42 DAF (Figure 4A). Lowest expression was detected for Class III *GmACBP5* and *GmACBP6* at 10–13 and 14–17 DAF (Figure 4A). *GmACBP5* lacked expression throughout every stage of seed development while *GmACBP6* exhibited low expression from 21 DAF (Figure 4A). Class III *GmACBP7* and *GmACBP8* both showed different expression profiles during seed development (Figure 4A). At 10–13 DAF, *GmACBP7* was highly expressed while *GmACBP8* expression was highest at 14–17 DAF. In Class IV, the expression profiles for all members were similar and *GmACBP10* and *GmACBP11* patterns were identical to each other (Figure 4A).

*GmACBP* expression in developing pods is shown in Figure 4A. Class I again showed very high expression at all three developmental stages. The expression of *GmACBP1* was notably similar across the three stages while *GmACBP2* expression was highest in 14–17 DAF pod shells (Figure 4A). Class II *GmACBP3* showed an upward trend as the pod shells matured in contrast to *GmACBP4* (Figure 4A). For Class III *GmACBP5* and *GmACBP8* lowest expression was evident in seven DAF pod shells (Figure 4A). *GmACBP6* indicated highest expression at 14–17 DAF while *GmACBP7* appeared constant across the different stages (Figure 4A). Class IV *GmACBP9*, *GmACBP10* and *GmACBP11* displayed different expression patterns at all three stages of pod development (Figure 4A).

Figure 4B shows *GmACBP* expression in young leaves, flowers, roots, and nodules. Class I *ACBP*s are highly expressed in young leaves and roots. Of the four organs, *GmACBP1* showed strongest expression in roots and weakest in young leaves while *GmACBP2* was similarly expressed in roots, nodules, and flowers, but displayed lowest expression in young leaves (Figure 4B). Class II *GmACBP3* and *GmACBP4* showed higher expression in nodules and roots than flowers and young leaves. Members of Class III *GmACBP5, GmACBP6, GmACBP7*, and *GmACBP8* projected divergent expression patterns in young leaves, flowers, nodules, and roots (Figure 4B). *GmACBP5* expression was greatest in roots but very low in young leaves, flowers, and nodules. In contrast, the expression of *GmACBP6* was highest in roots and well expressed in flowers. *GmACBP7* and *GmACBP8* shared greatest expression in the nodules. Class IV *GmACBP9*, *GmACBP10*, and *GmACBP11* showed about similar expression patterns in all four organs with highest expression in young leaves and roots (Figure 4B).

### *GmACBP* Expression in Response to Abiotic Stress

**FIGURE 5:**
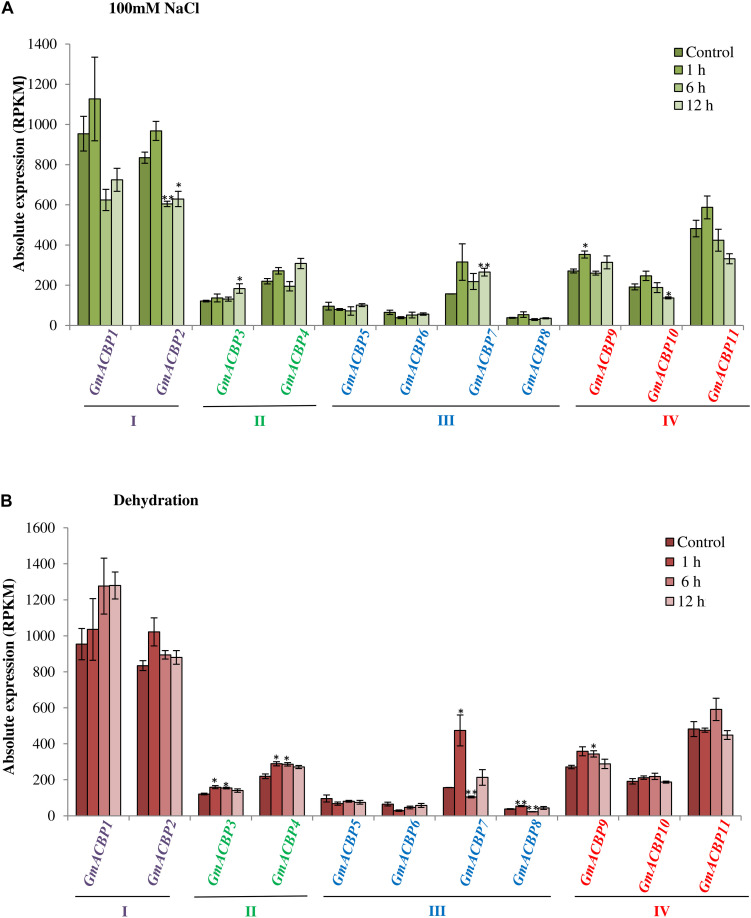
*GmACBP* expression in response to salinity and dehydration stress. Expression profiles were retrieved from transcriptomic data generated by Belamkar et al. (2014) accessible at SoyBase (https://www.soybase.org/) with the identifier GSE57252. **(A)** Salinity stress was imposed on Williams 82 soybean seedlings of V1 stage by Belamkar et al. (2014) by treating the plants with 100 mM NaCl solution at 1, 6, and 12 h. **(B)** The dehydration treatment was conducted by exposing the seedlings in the air with less water for 1, 6, and 12 h (Belamkar et al., 2014). Root tissues of five plants for each treatment were used for the RNA-seq (Belamkar et al., 2014). *GmACBP* classes are shown in different colors. The expression pattern was plotted based on the average expression value of three plants. Error bars indicate the standard error of mean for each sample. Control and treatment groups were compared using Student’s *t*-test. Statistically significant difference (**P < 0.01, *n* = 3; *P < 0.05, *n* = 3) is indicated.

**FIGURE 6:**
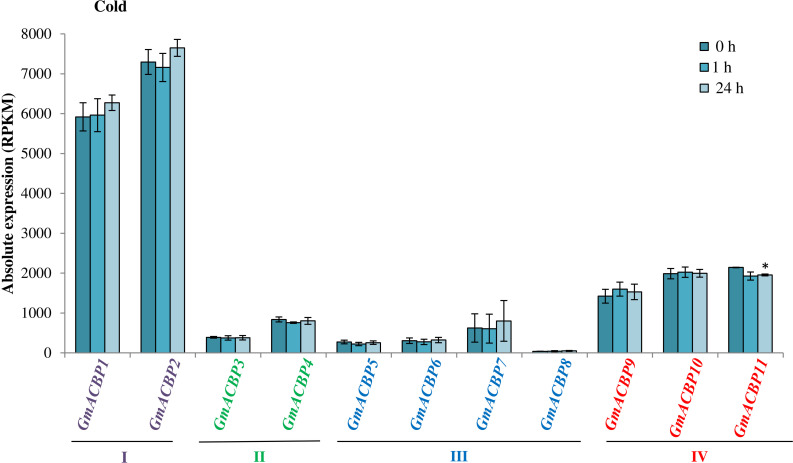
*GmACBP* expression profile following cold stress. The expression data were taken from SoyBase (https://www.soybase.org/) (GSE117686) based on the RNA-seq transcriptome generated by Robison et al. (2019). Two-week-old Williams 82 soybean seedlings were exposed to 4°C for 0, 1, and 24 h (Robison et al., 2019). RNA-seq was generated by harvesting the cold-treated unifoliate leaves of more than six plants per biological replicates of three (Robison et al., 2019). The control (0 h) was not subject to treatment at 4°C (Robison et al., 2019). *GmACBP* classes are represented by different colors. The expression profile was charted based on the average expression value of three plants. Error bars indicate the standard error of mean for each sample. Control and treatment groups were compared using Student’s *t*-test. Statistically significant difference (*P < 0.05, *n* = 3) is indicated.

Figure 5A shows *GmACBP* expression when subjected to salinity stress. Class I *GmACBP2* expression decreased within 6 h of treatment (Figure 5A). The expression of Class II *GmACBP3* was higher than the control after 12 h of salt treatment (Figure 5A). Class III *GmACBP7* expression pattern was different from *GmACBP5, GmACBP6* and *GmACBP8* because it appeared higher than the control within 12 h (Figure 5A). In Class IV, *GmACBP10* showed lower expression than the control at 12 h while *GmACBP9* expression was higher than the control at 1 h (Figure 5A). Figure 5B reports on *GmACBP* expression in response to dehydration and Class II *GmACBP3* and *GmACBP4* portrayed a similar expression pattern in which expression slightly increased and then was kept constant within 12 h (Figure 5B). Class III *GmACBP7* expression was upregulated within 1 h but after 6 h, expression dipped lower than the control (Figure 5B). Class IV *GmACBP9* displayed higher expression than the control after 6 h dehydration treatment (Figure 5B). Figure 6 shows that during cold stress no difference in expression was detected between the control and *GmACBPs*, with the exception of Class IV *GmACBP11* of which expression was lower than the control at 24 h.

### *GmACBP* Expression in Response to Biotic Stress

**FIGURE 7:**
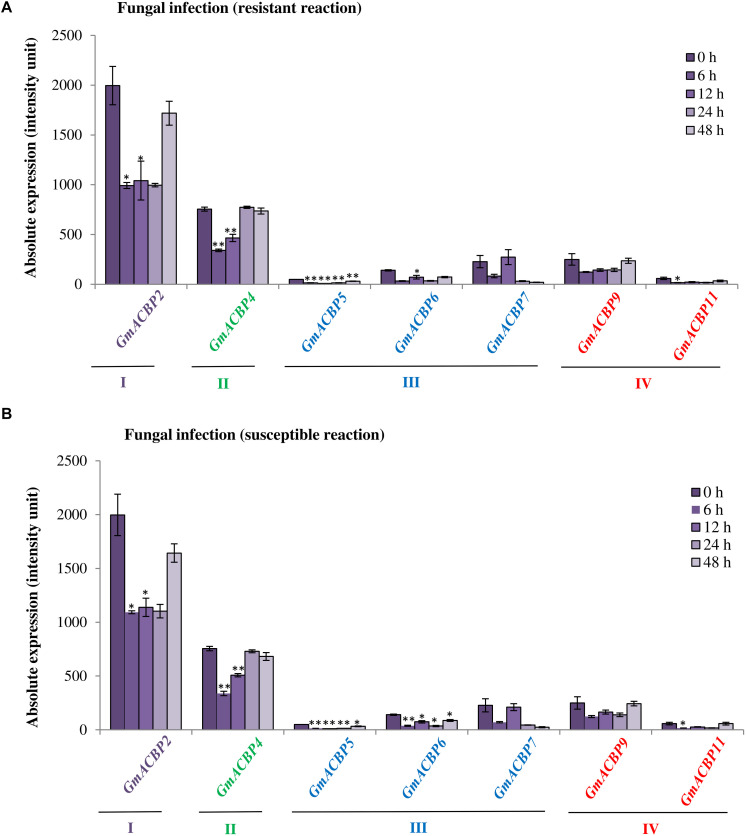
*GmACBP* expression in response to fungal infection by two different *Phakopsora pachyrhizi* strains. Expression data were extracted from the microarray analysis by Gregory Alvord et al. (2007) available in SoyBase (https://www.soybase.org/) with identifier (GDS3230). Isolates of *P. pachyrhizi* used in the microarray analysis by Gregory Alvord et al. (2007) produced either resistant or susceptible physiological response in the soybean host. **(A)** The *P. pachyrhizi* strain that produced resistant reaction was Hawaii 94-1, while **(B)** Taiwan 80-2 caused a susceptible reaction (Gregory Alvord et al., 2007). Microarray data were generated from RNA isolation of infected leaves from 0 to 48 h, and expression was plotted based on the average of three biological replicates. *GmACBP* classes are displayed in different colors. Error bars indicate the standard error of mean for each sample. Control and treatment groups were compared using Student’s *t*-test. Statistically significant difference (**P < 0.01, *n* = 3; *P < 0.05, *n* = 3) is indicated.

In microarray analysis of *GmACBP* expression during fungal *P. pachyrhizi* infection (Figure 7), only the expression of seven out of 11 was detected, albeit all classes were represented (Gregory Alvord et al., 2007) including Class I (*GmACBP2*), Class II (*GmACBP4*), Class III (*GmACBP5, GmACBP6* and *GmACBP7*), and Class IV (*GmACBP9* and *GmACBP11*). Similar expression patterns were observed for the seven *GmACBP*s in both susceptible- and resistant-reactions (Figures 7A,B). The expression of Class I *GmACBP2*, Class II *GmACBP4*, Class III *GmACBP5* and *GmACBP6*, and Class IV *GmACBP11* showed a reduction after 6 h (Figures 7A,B). For Class III *GmACBP5* and *GmACBP6*, expression was lower than the control up to 48 h (Figures 7A,B).

**FIGURE 8:**
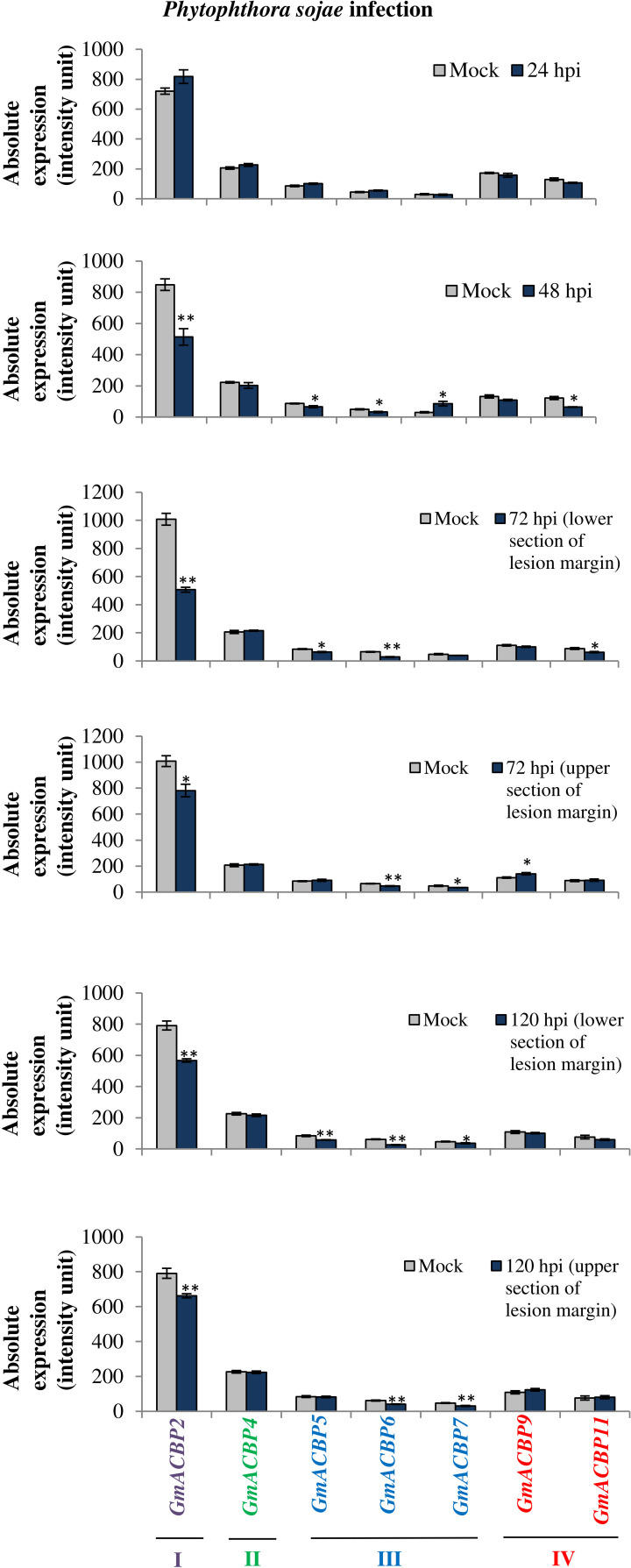
*GmACBP* expression in response to *Phytophthora sojae*. The data were plotted based on the microarray analysis performed by Wang et al. (2010) available at SoyBase (https://www.soybase.org/) with identifier GDS3244. Inoculation of *P. sojae* by Wang et al. (2010) was conducted by injuring the seedling tap root with a 5 mm wound using a scalpel and at 24 and 48 hpi the samples were taken at the inoculation site, while at 72 and 120 hpi, they were derived from 7.5 mm below and above the lesion margin of the seedlings. *GmACBP* classes are represented in different colors. The expression profile was generated based on the average of four biological replicates (Wang et al., 2010). Error bars indicate the standard error of mean for each sample. Control and treatment groups were compared using Student’s *t*-test. Statistically significant difference (**P < 0.01, *n* = 4; *P < 0.05, *n* = 4) is indicated.

Figure 8 portrays the expression of seven *GmACBPs* in response to *P. sojae.* Class I *GmACBP2* expression was significantly downregulated at 48, 72, and 120 hpi from the mock (Figure 8). Class III *GmACBP5, GmACBP6* and *GmACBP7* exhibited a reduction at 48, 72, 120 hpi (Figure 8). Class IV *GmACBP9* showed a slight increase over the mock at 72 hpi (upper section of lesion margin) while *GmACBP11* was downregulated at 48 and 72 hpi (lower section of lesion margin).

### *GmACBP* Expression in Soybean Root Nodules

**FIGURE 9:**
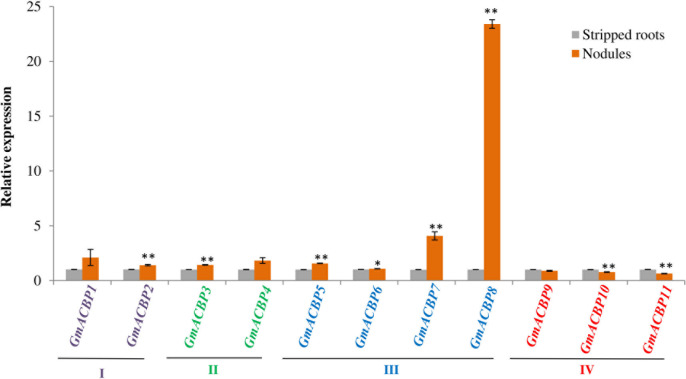
Quantitative real-time PCR of *GmACBP* expression in root nodules. The control for the experiment consisted of stripped roots, the main and lateral root regions after the removal of nodules following Wang Q. et al. (2020). The expression level was normalized to the housekeeping gene, *F-BOX PROTEIN2.* qRT-PCR analysis was performed in two biological replicates. A similar expression pattern was also observed in the second biological replicate. Error bars indicate the standard error of mean, *n* = 3. Control and target groups were compared using the Student’s *t-*test with statistically significant difference between the two groups as indicated (**P < 0.01; *P < 0.05).

Analysis by qRT-PCR on *GmACBP* expression in soybean root nodules showed that the expression of at least one member in each GmACBP class differed from the stripped root control (Figure 9). Expression of Class I *GmACBP2* and Class II *GmACBP3* were slightly higher in the nodules. While, the expression of Class IV *GmACBP10* and *GmACBP11* decreased in the nodules (Figure 9). For Class III, high expression in the nodules displayed by *GmACBP7* and *GmACBP8* corresponded to the expression pattern generated from RNA-seq (Severin et al., 2010) in which similar high expression occurred for *GmACBP7* and *GmACBP8* (Figure 4B). Such expression was again observed in qRT-PCR analysis of the second biological replicate and Class III *GmACBP8* exhibited very high expression in the nodules in comparison to the stripped roots (Supplementary Figure 1).

## Discussion

### Eleven GmACBPs Map Into Four ACBP Classes

All ACBP homologs in soybean could be classified into four classes similar to non-leguminous species Arabidopsis and rice. The classification of *GmACBP* members into the four respective plant ACBP classes had previously been displayed in phylogenetic analysis (Meng et al., 2011). Class I comprises small (10-kDa of ∼92 amino acids) proteins that contain only a single ACB domain. Class II consists of large ACBPs with additional functional motifs, ankyrin repeats at the *C*-terminus and transmembrane domain at the *N*-terminus (Li and Chye, 2003). Class III is defined by large (∼39.3 kDa) ACBPs in which the ACB domain resides at the *C-*terminus (Leung et al., 2006). Class IV is characterized by the ACB domain at the *N*-terminus and presence of kelch motif at the middle region of the protein (Leung et al., 2004). Genome-wide mining of ACBPs in several plant species has shown that at least one copy has been identified in each class indicating that plant ACBPs play non-redundant roles (Meng et al., 2011). On top of that, in soybean, the Class III GmACBP7 was predicted to contain an additional DnaJ domain which has not been reported in other plant ACBPs. DnaJ proteins are co-chaperone that interacts with HSP70 for activating its function in protein folding (Pulido and Leister, 2018). The discovery of the DnaJ domain in GmACBP7 is deemed interesting as DnaJ proteins are characterized by the J-domain where HSP70 interaction occurs, with the addition of a zinc-finger domain and/or *C*-terminal substrate-binding domain (Pulido and Leister, 2018).

### ACB Domain Sequence Conservation Amongst Arabidopsis, Rice, and Soybean

Alignment of ACB domains within the same classes revealed high conservation amongst Arabidopsis, rice and soybean. This may be explained by the selection pressure on *ACBP* genes in monocots and eudicots by which any deleterious mutation in *ACBP* will be eliminated to retain its biological function in binding acyl-CoA esters (Burton et al., 2005; Cvijović et al., 2018). Multiple protein sequence alignment of ACBPs from Arabidopsis, *Brassica napus*, rice, *Ricinus communis* (castor bean), man and cow showed that several amino acid residues are conserved in all, including YKQATVGP and KAKWDAW, which corresponded to the acyl-CoA-binding site (Engeseth et al., 1996). Considering the high homology amongst ACBP amino acid sequences, the 3D structure of the ACB domain in GmACBPs was predicted based on published ACBP structures from other plants or organisms. The structure of ACBPs as of bovine ACBP determined by nuclear magnetic resonance spectroscopy (Andersen et al., 1991), and those from man (Taskinen et al., 2007), *Plasmodium falciparum* (van Aalten et al., 2001) and rice (Guo et al., 2017; Jin et al., 2020) showed that each ACBP is made of four α-helices displayed in an up-and-down configuration. In rice, the acyl-CoA ester C18:3-CoA was shown to bind to Class I OsACBP2 in a pocket formed by the two helical structures and the fatty acyl group of the ester was accommodated in the region of hydrophobic residues (Jin et al., 2020).

### Subcellular Localization of GmACBPs

Subcellular localization of GmACBPs as analyzed using two different online tools, TargetP 1.1 (Emanuelsson et al., 2000) and PSORT (Nakai and Horton, 1999), revealed variation in the results, which may have arisen from different methods applied in each prediction. TargetP 1.1 specifically analyzes the *N*-terminal amino acid sequence for targeting into chloroplast, mitochondrion, ER/Golgi/secreted and ‘other’ (Emanuelsson et al., 2000). Meanwhile, PSORT takes into account the full amino acid sequence and also its organism origin, which is either prokaryotes, yeasts, animals or plants. In addition, PSORT analyzes various sequence features that range more than just the chloroplast or mitochondrion including the peroxisomal-targeting signal, ER-lumen-retention signal, nuclear-localization signals and vacuolar-targeting signal (Nakai and Horton, 1999) which may result in a more diverse prediction than TargetP 1.1.

Classes I and IV GmACBPs were deemed to be cytosolic using PSORT similar to Arabidopsis Class I AtACBP6 and Class IV AtACBP4 and AtACBP5, which had been verified by western blot analysis of subcellular protein fractionation, immuno-electron microscopy and confocal laser scanning microscopy of autofluorescence-tagged AtACBPs (Chen et al., 2008; Xiao et al., 2008). For Class II GmACBPs, both members were predicted to localize at the plasma membrane, similarly to Arabidopsis Class II as substantiated by transient expression of AtACBP-GFP fusion in onion epidermal cells and immuno-electron microscopy (Li and Chye, 2003), and expression of DsRed-AtACBP1 fusion protein in transgenic Arabidopsis (Lung et al., 2017, 2018). In rice, Class II OsACBP4 was validated to be localized at the plasma membrane and ER by confocal laser scanning microscopy of transgenic rice roots expressing *OsACBP4pro:GFP* (Liao et al., 2020). Extracellular targeting of Class III GmACBP5 according to PSORT is consistent with findings on Class III Arabidopsis AtACBP3 and rice OsACBP5, which were localized to the apoplast (Leung et al., 2006; Liao et al., 2020).

### *GmACBP* Expression in Different Organs

High expression of Class I *GmACBP* in the flowers, roots, nodules and young leaves (Figure 4) indicated an expression profile suitable to housekeeping functions of Class I *ACBPs* (Du et al., 2016). Interestingly, Class III *GmACBP7* and *GmACBP8* displayed high expression in the nodules as shown by RNA-seq (Figure 4B) and experimentally validated by qRT-PCR analysis (Figure 9) suggesting their involvement in nodulation. Expression of *GmACBPs* in nodules is unique for soybean as this specialized organ does not occur in Arabidopsis or rice (Stacey, 2007). Nodules are organs developed from symbiosis in relation with rhizobia for atmospheric nitrogen fixation (Ferguson, 2013). An understanding on how GmACBPs can regulate nodulation would be essential as this symbiosis process is a good alternative to nitrogen-based fertilizers which contribute to air pollution in its production and the product itself can be very costly (Crutzen et al., 2016).

The seed development of a legume can be divided into three main phases, namely embryogenesis, maturation, and dormant/matured stages (Le et al., 2007). The embryogenesis stage is further categorized into globular, heart and cotyledon stages. In general, the early stage in seed development is for cell division and cell differentiation of embryo axis and cotyledons (Le et al., 2007). After embryogenesis, the seed will enter maturation stage which is divided into early-, mid-, and late-stage (Le et al., 2007). The seed-filling stage is considered to be the most crucial phase in the seed development (Le et al., 2007) because this is the stage where the accumulation of reserves such as lipids, carbohydrate and protein occurs (Le et al., 2007). If disruption takes place during the accumulation process, it will negatively affect yield (Sehgal et al., 2018). The final stage in seed development is the dormant stage during which the seeds lose water and stay dormant until favorable conditions are available for germination (Sehgal et al., 2018). The seeds that Severin et al. (2010) collected are classified into cotyledon stage (10–13 and 14–17 DAF), early-maturation 1 (21, 25, and 28 DAF), early-maturation 2 (35 DAF) and mid-maturation (42 DAF) stage according to plant ontology provided by SoyBase^13^.

High expression of Class I *GmACBP1* and *GmACBP2* in developing seeds suggests that Class I GmACBPs play important roles during seed development. Their expression remained high during early maturation which was the beginning of seed-filling stage. Studies conducted on Class I ACBPs in Arabidopsis (Guo et al., 2019b), *Helianthus annuus* (sunflower) (Aznar-Moreno et al., 2016), rice (Guo et al., 2019a), *Elaeis guineensis* (oil palm) (Amiruddin et al., 2019), and *B. napus* (Yurchenko et al., 2009, 2014) suggested their involvement in seed oil biosynthesis. Comparative transcriptomic analysis followed by qRT-PCR on developing embryos and seed coats of *B. napus*, showed that the Class I *BnACBP6* displayed the highest of all *BnACBP* expression throughout seed development suggesting it plays a potential role in oil accumulation (Liao et al., 2019). In oil palm and sunflower, the expression of their Class I *ACBPs* in the seeds was highest during the oil accumulation period (Aznar-Moreno et al., 2016; Amiruddin et al., 2019). In transgenic rice, the overexpression of Class I *OsACBP2* increased grain size and oil content (Guo et al., 2019a). Knockout of *atacbp6* affected seed oil content and composition (Guo et al., 2019b), leading to reduction in seed weight (Hsiao et al., 2014). This is not surprising given the main components for the biosynthesis of oil in seeds are glycerol-3-phosphate (G3P) and acyl-CoA esters, which act as substrates for the acylation of G3P catalyzed by acyltransferases to form triacylglycerol (TAG) (Bates et al., 2013). *In vitro* studies on recombinant *B. napus* Class I ACBP (rBnACBP) have already shown that it can stimulate the activity of acyltransferases involved in TAG synthesis (Brown et al., 2002).

### *GmACBP* Expression in Response to Abiotic Stress

RNA-seq analysis for salinity and drought stress response (Figures 5A,B) showed that the increase in Class II *GmACBP3* expression is similar to Arabidopsis and rice (Du et al., 2013; Chen et al., 2018; Guo et al., 2020) but Class III *GmACBP7* was also induced. In Arabidopsis, it has been shown that Class II AtACBPs play essential roles in both drought and salinity stress (Du et al., 2013; Chen et al., 2018). *AtACBP1* expression was induced greater than five-fold after 48 h NaCl treatment (Chen et al., 2018). Studies using *AtACBP1*-overexpressors, *atacbp1*, and *AtACBP1*-complemented lines in response to different salinity concentrations revealed that AtACBP1 is involved in the abscisic acid (ABA)-signaling pathway and interacted with ABA-RESPONSIVE ELEMENT BINDING PROTEIN1 (AREB1) through its ankyrin repeats to regulate salinity responses during seed germination and seedling development (Chen et al., 2018). Meanwhile, the expression of *AtACBP2* in the guard cells supported its role in the drought stress response through an ABA-mediated pathway in the production of reactive oxygen species (ROS) and *AtACBP2*-overexpression in transgenic Arabidopsis conferred drought tolerance (Du et al., 2013). In rice, Class II *OsACBP4* expression was induced by both salt and drought treatment (Meng et al., 2011). Furthermore, the overexpression of *OsACBP4* enhanced tolerance to salinity stress in transgenic rice and Arabidopsis (Guo et al., 2020). In this study, the Class III member *GmACBP7* was found to be more greatly induced by salinity and drought than the two Class II members, *GmACBP3* and *GmACBP4* (Figure 5), which makes soybean different from rice and Arabidopsis because in these species only Class II *ACBPs* have been reported to be upregulated after salinity or drought treatment. This may imply different roles played by GmACBPs in soybean stress responses.

When soybean seedlings were subjected to cold stress (Robison et al., 2019), the expression of most *GmACBPs* from Classes I, II, and III did not exhibit any difference between control and the cold-treated samples. In contrast, Arabidopsis Class II AtACBP1 and Class I AtACBP6 played a role in response to cold stress (Chen et al., 2008; Du et al., 2010). AtACBP1 was implicated in the cold stress response through the regulation of phospholipase Dα1 (PLDα1) and phospholipase Dδ (PLDδ) that were related to the changes in phosphatidylcholine (PC) and phosphatidic acid (PA) contents although *AtACBP1* expression was not cold-inducible (Du et al., 2010). Initial studies conducted on rosettes of 4-week-old Arabidopsis (Chen et al., 2008) and subsequently in the flowers of 5-week-old plants (Liao et al., 2014) revealed that the overexpression of *AtACBP6* conferred cold tolerance through different mechanisms in different organs that were related to the expression of *COLD-RESPONSIVE (COR)*-related, PC-related and monogalactosyldiacylglycerol (MGDG)-related genes. The expression of rice Class I ACBPs (*OsACBP1*, *OsACBP2*, and *OsACBP3*) was down-regulated in 12 h but at 24 h their expression had resumed to level similar to the control (Meng et al., 2011). In each of Arabidopsis, rice, and soybean, distinct patterns in Class I ACBP expression upon cold stress suggest that variation probably arose from differences in tissues used in each study. In rice, 2-week-old rice seedlings were used (Meng et al., 2011) while in soybean, RNA-seq data were generated from unifoliate 2-week-old soybean seedlings (Robison et al., 2019).

### *GmACBP* Expression in Response to Biotic Stress

Various strains of *P. pachyrhizi* can have specific host-strain reactions resulting in differential resistance reactions (Hartman et al., 2011). The strains used in Gregory Alvord et al. (2007), were Hawaii 94-1, an avirulent strain that produced resistant-reaction for the soybean host and Taiwan 80-2, a virulent strain that resulted in susceptible-reaction (Bonde et al., 2006). In the analysis of *GmACBP* expression toward different strains of *P. pachyrhizi*, there was a lack in distinct variation in expression between the susceptible and resistant reactions suggesting that *GmACBPs* may not function directly in producing these reaction types. Studies on identification on differentially expressed genes (Gregory Alvord et al., 2007) or accumulation of metabolites (Silva et al., 2020) between two conditions of susceptibility and resistance are expected to achieve an understanding of the molecular pathway for soybean resistance against the pathogen (Hartman et al., 2016).

In soybean, single dominant genes that conferred resistance toward *P. sojae* were identified as *Rps* genes and had been applied in breeding resistant cultivars (Gordon et al., 2007). However, some populations of *P. sojae* that surpass the resistance genes do exist (Dorrance et al., 2003). In a study by Wang et al. (2010), the cultivar Conrad used was a variety that conferred partial resistance toward the pathogen. Partial resistance was characterized by reduction in pathogen colonization and size of lesions (Mideros et al., 2007). Inoculation of cultivar Conrad with *P. sojae* revealed that most members of Classes I, III, and IV exhibited slight downregulation in expression from the control. This situation appeared to differ from ACBPs of other plants such as Arabidopsis, grapevine and rice, in which Class III ACBPs, AtACBP3 (Xiao and Chye, 2011), VvACBP (Takato et al., 2013), and OsACBP5 (Panthapulakkal Narayanan et al., 2020) respectively, respond to plant pathogens. In transgenic Arabidopsis, *AtACBP3*-overexpressors and *acbp3* showed contrasting reactions to biotrophic and necrotrophic pathogens. *AtACBP3*-overexpressors were resistant to infection by biotrophic pathogen *Pseudomonas syringae* pv. *tomato* DC3000, but was susceptible to infection by necrotrophic fungus *Botrytis cinerea* (Xiao and Chye, 2011). In contrast, transgenic rice *OsACBP5*-overexpressors displayed disease resistance toward biotrophic (*Xanthomonas oryzae*), hemibiotrophic (*Magnaporthe oryzae* and *Fusarium graminearum*) and necrotrophic (*Rhizoctonia solani* and *Cercospora oryzae*) pathogens *via* both jasmonic acid (JA)- and salicylic acid (SA)-mediated pathways (Panthapulakkal Narayanan et al., 2020). Also transgenic Arabidopsis overexpressing *V. vinifera* Class III ACBP was tolerant to *P. syringae* and *Colletotrichum higginsianum*, a pathogenic hemibiotrophic ascomycetous fungus (Takato et al., 2013).

## Conclusion

In this analysis of GmACBPs, their protein domain architecture and their sequences were demonstrated to be well conserved to ACBPs from non-leguminous plants such as Arabidopsis and rice. The analysis on putative *GmACBP* spatial expression at various organs and different developmental stages, along with expression in response to stress showed that GmACBPs may play important roles in nodule formation that would be unique from Arabidopsis and rice ACBPs.

## Data Availability Statement

The raw data supporting the conclusions of this article will be made available by the authors, without undue reservation.

## Author Contributions

NSA, S-CL, and M-LC designed the research. NSA analyzed the data and conducted qRT-PCR analysis. Z-HG and S-CL helped in data analyses. ZW, W-SY, and H-ML prepared RNA and cDNA from soybean root nodules and stripped roots. NSA and M-LC wrote the manuscript with contributions of all authors. All authors contributed to the article and approved the submitted version.

## Conflict of Interest

The authors declare that the research was conducted in the absence of any commercial or financial relationships that could be construed as a potential conflict of interest. The reviewer JH declared a past co-authorship with one of the authors M-LC to the handling editor.

## Footnotes

1 https://www.soybase.org/

2 http://bar.utoronto.ca/efpsoybean/cgi-bin/efpWeb.cgi

3 https://phytozome.jgi.doe.gov/pz/portal.html

4 http://blast.ncbi.nlm.nih.gov/Blast.cgi

5 https://www.ncbi.nlm.nih.gov/Structure/cdd/cdd.shtml

6 http://www.sbg.bio.ic.ac.uk/phyre2/

7 https://swissmodel.expasy.org/

8 https://www.ncbi.nlm.nih.gov/Structure/icn3d/full.html

9 https://www.geneious.com

10 http://www.cbs.dtu.dk/services/TargetP-1.1/

11 http://psort1.hgc.jp/form.html

12 https://www.soybase.org/expression/

13 https://www.soybase.org/ontology.php

## Supplementary Material

Supplementary Figure 1 Quantitative real-time PCR of *GmACBP* expression in root nodules in second biological replicate. The expression level was normalized to the housekeeping gene, *F-BOX PROTEIN2*. Error bars indicate the standard error of mean, *n* = 3. Control and target groups were compared using Student’s *t*-test. Statistically significant difference (^∗∗^*P* < 0.01; ^∗^*P* < 0.05) is indicated.

Supplementary Table 1 Gene-specific primers for qRT-PCR analysis of *GmACBP* in nodules.
