# Supplementary figures and images for: In silico Analysis of Acyl-CoA-Binding Protein Expression in Soybean
Source: Front Plant Sci. 2021 Apr 15;12:646938. doi: 10.3389/fpls.2021.646938 (PMC8082252; doi:10.3389/fpls.2021.646938)

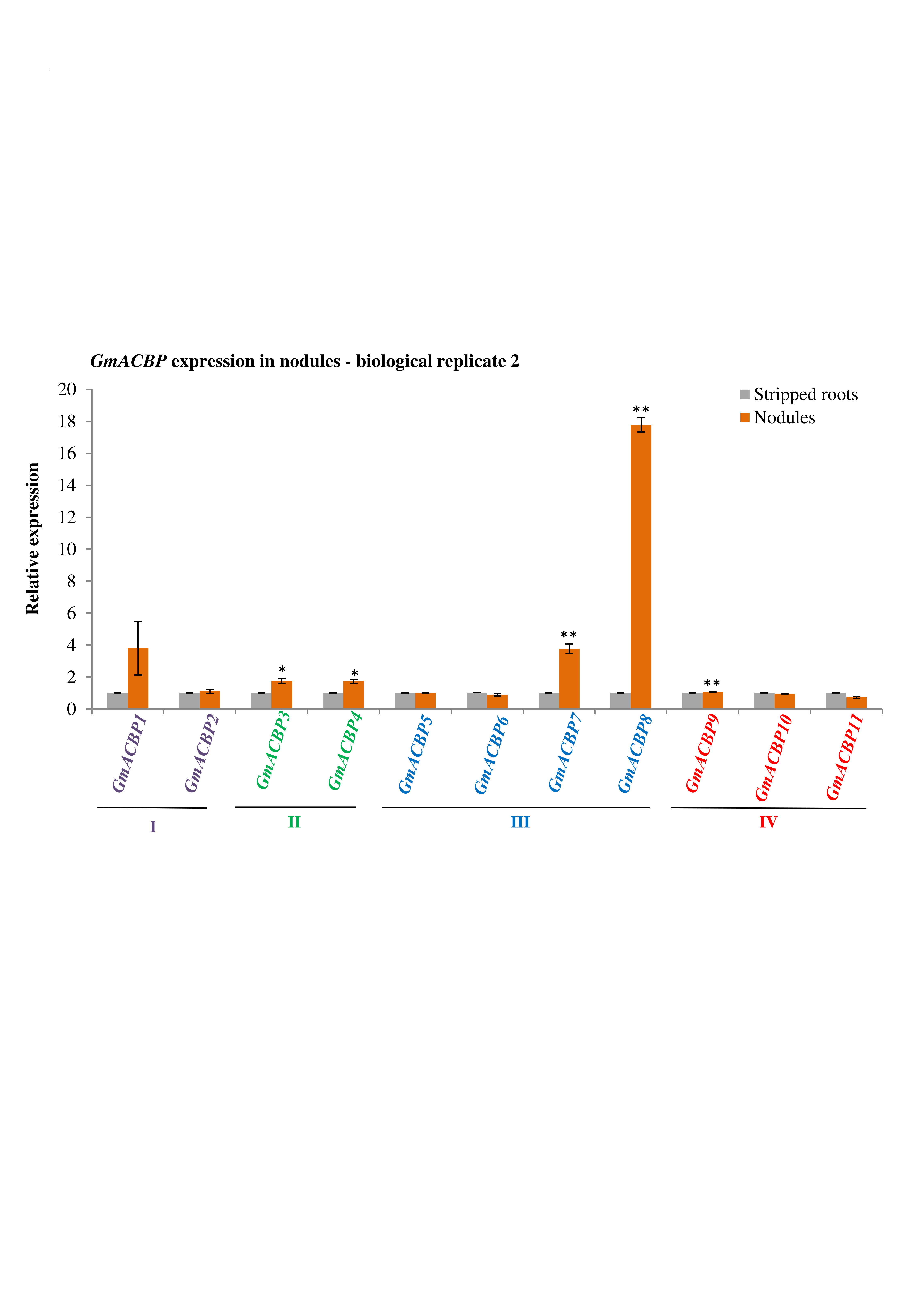

Supplement: Supplementary Figure 1 — Quantitative real-time PCR of GmACBP expression in root nodules in second biological replicate. The expression level was normalized to the housekeeping gene, F-BOX PROTEIN2. Error bars indicate the standard error of mean, n = 3. Control and target groups were compared using Student’s t-test. Statistically significant difference (∗∗P < 0.01; ∗P < 0.05) is indicated. [file Image_1.TIF]
