# Supplementary material for: In silico Analysis of Acyl-CoA-Binding Protein Expression in Soybean
Source: Front Plant Sci. 2021 Apr 15;12:646938. doi: 10.3389/fpls.2021.646938 (PMC8082252; doi:10.3389/fpls.2021.646938)
Supplement: Supplementary Table 1 — Gene-specific primers for qRT-PCR analysis of GmACBP in nodules. [file Table_1.DOCX]

**Table S1.** Gene-specific primers for qRT-PCR analysis of *GmACBP* in nodules

| Gene | Forward (5’ – 3’) | Reverse (5’ – 3’) |
| --- | --- | --- |
| *GmACBP1* | TCATTTGGACATGGACATGGACA | CATTCCAGGACGGCTGGT |
| *GmACBP2* | TAATTGGGACATGGTCATGCAGG | CATTCCCGGACGGCTGGT |
| *GmACBP3* | GAGGAATAAAAGTGGGGACA | CTTCCATTTGTGAGTCACTGC |
| *GmACBP4* | GAGGAATAAAAGCGGCGAC | CTTCCATTTGTGAGTCACTGC |
| *GmACBP5* | TCCTTATGATAATGCTAAACCGGC | TGCTAAGCGTCGGAATTCAA |
| *GmACBP6* | TCCTTATGATAATGCTAAACCGGC | CTTCCCTGCAGCGCAGTT |
| *GmACBP7* | TTGCCACCGAAGGGCCA | TTTCATGTTCACCTATTCCAGCAG |
| *GmACBP8* | TTGCGACGGAAGGGCTTT | TTTCATGTTCACTCATTCCAGCAG |
| *GmACBP9* | CAGAGACAAAGACTATTTCCACCC | ACTGCAGCTCCATGCTCATA |
| *GmACBP10* | GCAGGCCATGATTCTAATCCAC | CGAACTGATTGGAGCTCCTTG |
| *GmACBP11* | CCAGAGATAAAAACCATTTCCACTG | ACTGCAGCTCCATGCTCATA |
| *F-BOX PROTEIN2* | TGAGAAAGCTGTTGAGGATT | GATTGCTCTTAAATCCATGC |
